# Identification of Quantitative Trait Nucleotides and Development of Diagnostic Markers for Nine Fatty Acids in the Peanut

**DOI:** 10.3390/plants13010016

**Published:** 2023-12-20

**Authors:** Juan Wang, Haoning Chen, Yuan Li, Dachuan Shi, Wenjiao Wang, Caixia Yan, Mei Yuan, Quanxi Sun, Jing Chen, Yifei Mou, Chunjuan Qu, Shihua Shan

**Affiliations:** 1 Shandong Peanut Research Institute, Qingdao 266100, China; wangjuan_1984@163.com (J.W.);; 2 National Bioinformatics Infrastructure Sweden, Science for Life Laboratory, Lund University, 22100 Lund, Sweden; 3 Department of Immunotechnology, Lund University, Medicon Village, 22100 Lund, Sweden; 4 Qingdao Academy of Agricultural Sciences, Qingdao 266100, China

**Keywords:** peanut oil, fatty acids, GC-MS, GWAS, PARMS

## Abstract

The cultivated peanut (*Arachis hypogaea* L.) is an important oilseed crop worldwide, and fatty acid composition is a major determinant of peanut oil quality. In the present study, we conducted a genome-wide association study (GWAS) for nine fatty acid traits using the whole genome sequences of 160 representative Chinese peanut landraces and identified 6-1195 significant SNPs for different fatty acid contents. Particularly for oleic acid and linoleic acid, two peak SNP clusters on Arahy.09 and Arahy.19 were found to contain the majority of the significant SNPs associated with these two fatty acids. Additionally, a significant proportion of the candidate genes identified on Arahy.09 overlap with those identified in early studies, among which three candidate genes are of special interest. One possesses a significant missense SNP and encodes a known candidate gene *FAD2A*. The second gene is the gene closest to the most significant SNP for linoleic acid. It codes for an MYB protein that has been demonstrated to impact fatty acid biosynthesis in *Arabidopsis*. The third gene harbors a missense SNP and encodes a JmjC domain-containing protein. The significant phenotypic difference in the oleic acid/linoleic acid between the genotypes at the first and third candidate genes was further confirmed with PARMS analysis. In addition, we have also identified different candidate genes (i.e., *Arahy.ZV39IJ*, *Arahy.F9E3EA*, *Arahy.X9ZZC1,* and *Arahy.Z0ELT9*) for the remaining fatty acids. Our findings can help us gain a better understanding of the genetic foundation of peanut fatty acid contents and may hold great potential for enhancing peanut quality in the future.

## 1. Introduction

The cultivated peanut (*Arachis hypogaea* L.) is an important oil crop worldwide, and China contributes to approximately 40% of global peanut production (http://www.fao.org, accessed on 2 June 2022). In China, the peanut accounts for half of the total oil crop production, making it the leading oil crop. Fatty acid composition is a significant factor that determines the flavor, shelf life, and nutritional quality of peanuts. In peanuts, fatty acids mainly consist of three unsaturated fatty acids (oleic acid/C18:1, linoleic acid/C18:2, and gadoleic acid/C20:1) and six saturated fatty acids (palmitic acid/C16:0, stearic acid/C18:0, arachidic acid/C20:0, behenic acid/C22:0, lignoceric acid/C24:0, and heptadecanoic acid/C17:0). Among those fatty acids, oleic acid and linoleic acid account for up to 80% of the total fatty acid content in peanuts [1]. As a monounsaturated fatty acid, oleic acid is considered the most desirable fatty acid due to its potential to inhibit tumor growth, lower blood cholesterol levels, and combat inflammatory diseases [2,3,4].

In plants, the *de novo* synthesis of fatty acids starts with acetyl-CoA, which undergoes a series of reactions to form palmitic acid (16:0), a 16-carbon saturated fatty acid [5,6]. Palmitic acid is subsequently modified to produce various other fatty acids. For instance, it is first elongated to form stearic acid (C18:0), which can then be desaturated to oleic acid (C18:1) by stearoyl-acyl carrier protein desaturase in the plastids. Oleic acid may, in turn, be further desaturated to linoleic acid (C18:2), either by fatty acid desaturase 6 (FAD6) in the plastids or by FAD2 in the endoplasmic reticulum (ER). Linoleic acid can be even further desaturated into γ-linolenic acid, either by FAD3 in the ER or by FAD7/FAD8 in plastids [7]. Therefore, it is possible to boost the oleic acid content of peanuts when oil is the preferred product by increasing the inflow from acetyl-CoA and/or reducing the outflow to linoleic acid. The latter can be achieved through mutations that inactivate the FAD2 desaturase [8,9,10]. However, FAD2 mutants may compromise other important agronomic traits, such as stress resistance [11]. Hence, discovering novel QTLs for genetic improvement is necessary, and a great deal of effort has been made [9,10,12,13]. However, due to the complexity of the genetic underpinning of these quantitative traits, there is still much work to be done. Furthermore, the genetic basis of several other peanut fatty acids remains largely undiscovered.

In the present study, we focused on 160 Chinese peanut landraces with rich genetic variation [14,15] and used a gas chromatograph-mass spectrometer (GC-MS) to accurately determine their relative fatty acid contents. Based on the acquired fatty acid contents, we conducted genome-wide association studies (GWASs) to identify SNPs associated with different fatty acids, aiming to enhance our understanding of the genetic basis of peanut fatty acids.

## 2. Results

### 2.1. Characterization and Distribution of SNPs in the Peanut Genome

**Figure 1:**
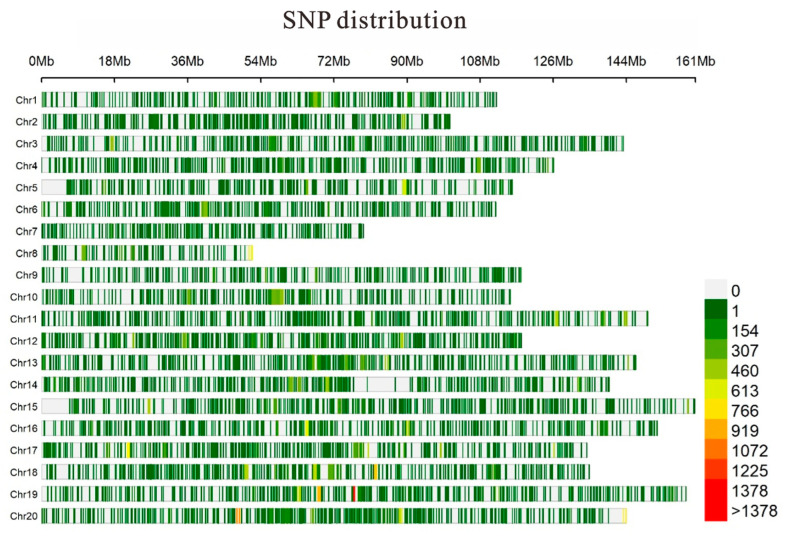
Distribution of single-nucleotide polymorphisms (SNPs) on each of the 20 chromosomes of the cultivated peanut. The top scale indicates chromosome location (in Mb), with color representing SNP density (the number of SNPs per window).

A total of 116,443 high-quality genome-wide SNPs was obtained (Figure 1; Table S1). Most of the identified SNPs were found in intergenic regions (79.5%), while those in the exonic, intronic, and up- and downstream regions of the annotated gene account for 1.6%, 3.2%, and 15.6% of the total SNPs, respectively. “A/G” is the most abundant SNP, accounting for 34.10% of the total SNPs, followed by “C/T” (33.60%). “A/C”, “G/T”, “A/T”, and “C/G” account for 8.71%, 8.94%, 9.48%, and 5.17% of the total SNPs, respectively.

### 2.2. Fatty Acid Determination

**Figure 2:**
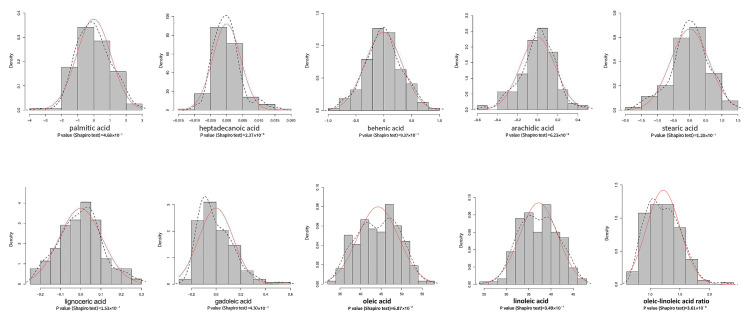
Frequency distribution of the studied fatty acid traits. *X*-axis: BLUP values of the studied traits; black dotted line: kernel density plot; red line: normal distribution.

Peanuts contain nine main fatty acids, listed in decreasing order of relative concentrations: oleic acid (C18:1), linoleic acid (C18:2), palmitic acid (C16:0), stearic acid (C18:0), behenic acid (C22:0), arachidic acid (C20:0), gadoleic acid (C20:1), lignoceric acid (C24:0), and heptadecanoic acid (C17:0). Among these fatty acids, oleic acid accounts for 28.76–52.02% of the total oil content, linoleic acid accounts for 23.06–45.84%, palmitic acid accounts for 10.94–19.67%, and lignoceric acid accounts for 0.20–1.20% (Table 1). The heritabilities (H^2^) of oleic acid and linoleic acid were the highest among the nine fatty acids (0.92), while that of heptadecanoic acid was the lowest (0.71) (Table 1). Among the studied varieties, the irregular types that were hybrids among the four botanical varieties had the highest oleic acid/linoleic acid ratio, while var. *fastigiata* had the lowest ratio. Based on BLUP values, all the studied fatty acids followed a normal distribution (Figure 2).

### 2.3. Phenotypic Correlation

**Figure 3:**
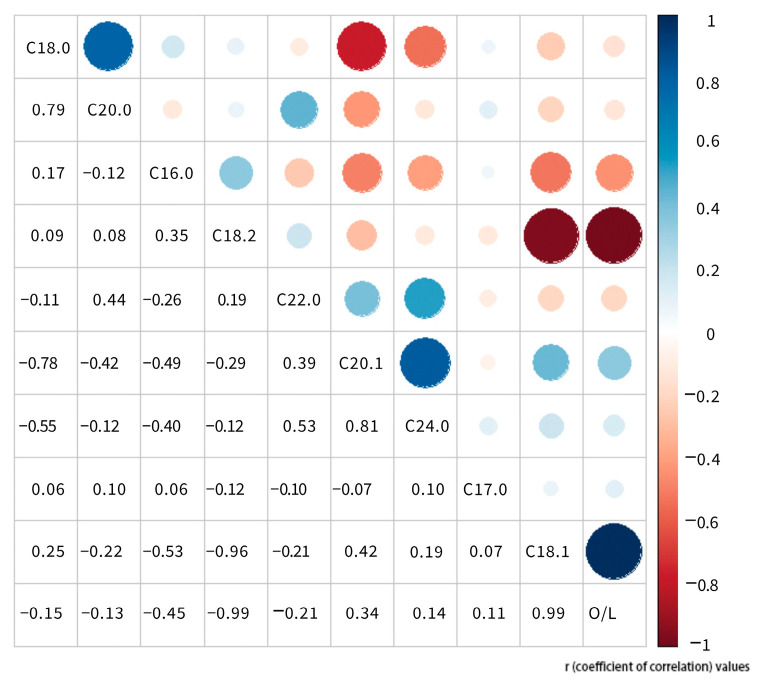
Correlation between the studied fatty acid traits. Dot color and size both represent the degree of correlation. These numbers represent r (coefficient of correlation) values.

Both positive and negative correlations were detected among the studied fatty acids. The most negative correlation was between oleic acid (C18:1) and linoleic acid (C18:2) (*r* = −0.96), followed by stearic acid (C18:0) and gadoleic acid (C20:1) (*r* = −0.78). The most positive correlation was between gadoleic acid (C20:1) and lignoceric acid (C24:0) (0.81), followed by stearic acid (C18:0) and arachidic acid (C20:0) (*r* = 0.79) (Figure 3). A lower negative correlation was observed between palmitic acid (C16:0) and oleic acid (C18:1) (*r* = −0.53) and between stearic acid (C18:0) and lignoceric acid (C24:0) (*r* = −0.55). A lower positive correlation was found between behenic acid (C22:0) and lignoceric acid (C24:0) (*r* = 0.53). The absolute values of the correlation coefficients between the other fatty acids are below 0.5.

### 2.4. Genome-Wide Association Studies (GWASs) in Peanuts

**Figure 4:**
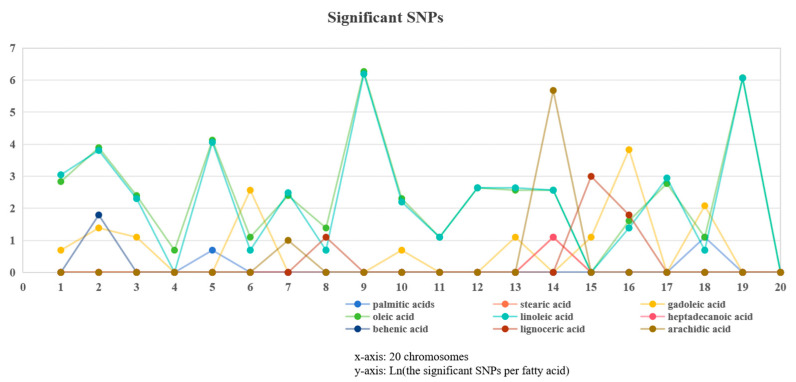
The significant SNP density on each chromosome for the nine studied fatty acids.

**Figure 5:**
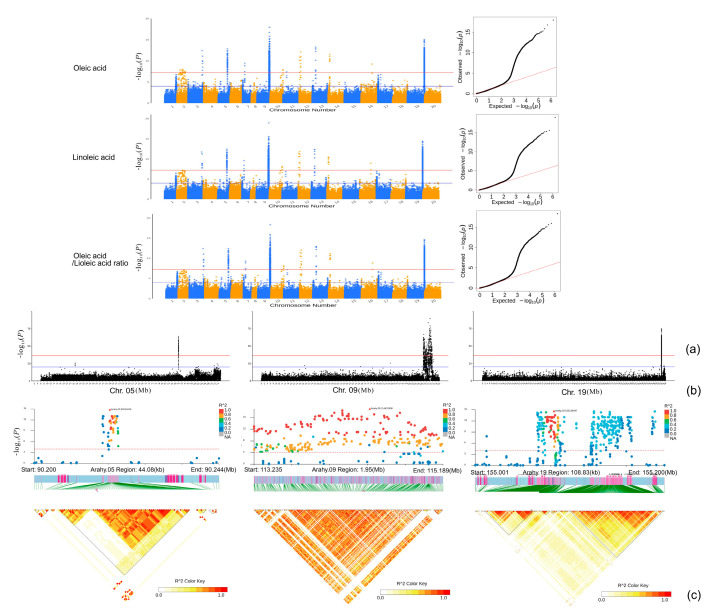
GWAS results for oleic acid (C18:1), linoleic acid (C18:2), and the oleic/linoleic acids (O/L) ratio. (**a**) Manhattan plots and QQ plots. The blue and red horizontal lines represent, respectively, the significance thresholds of −log_10_(*p*) = 5 and −log_10_(*p*) = 6. (**b**) Manhattan plots for Arahy.05, Arahy.09, and Arahy.19. (**c**) Local Manhattan plots (top) and LD heatmaps (bottom) at the three candidate regions 90.200–90.244 Mb (Arahy.05), 113.235–115.189 Mb (Arahy.09), and 155.091–155.200 Mb (Arahy.19).

**Figure 6:**
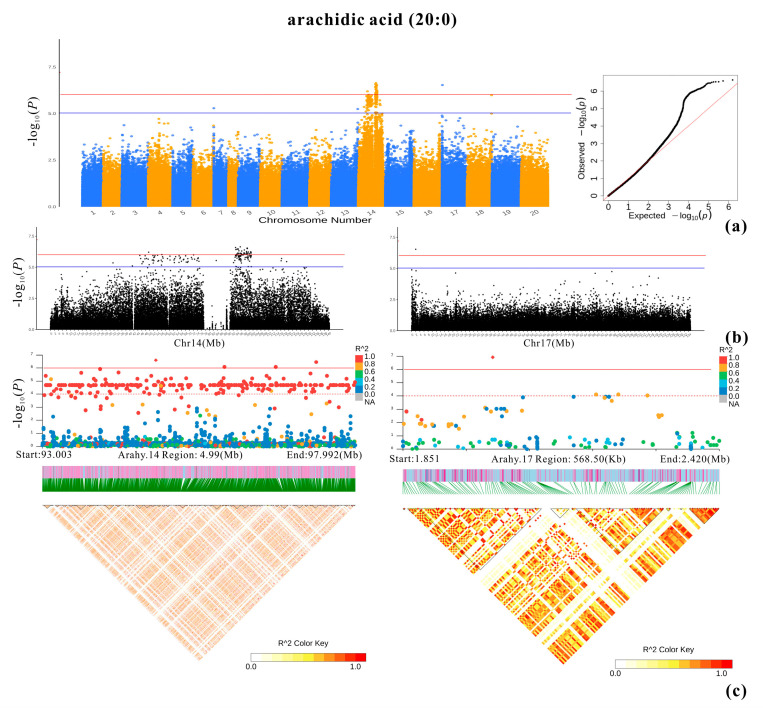
GWAS result for arachidic acid. (**a**) Manhattan plot and QQ plot. The blue and red lines represent, respectively, the significance thresholds of −log_10_(*p*) = 5 and −log_10_(*p*) = 6. (**b**) Local Manhattan plots of Arahy.14 and Arahy.17. (**c**) Local Manhattan plots (top) and LD heatmaps (bottom) at regions 93.003–97.992 Mb (Arahy.14) and 1.857–2.420 Mb (Arahy.17).

**Figure 7:**
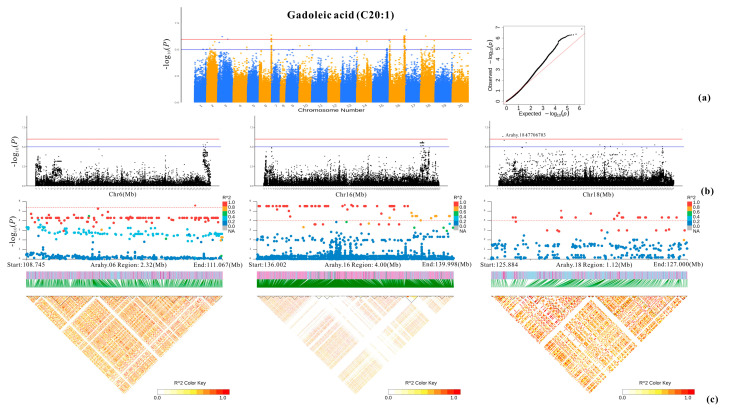
GWAS result for gadoleic acid. (**a**) Manhattan plot and QQ plot. The blue and red lines represent, respectively, the significance thresholds of −log_10_(*p*) = 5 and −log_10_(*p*) = 6. (**b**) Local Manhattan plots on Arahy.06, Arahy.16, and Arahy.18. (**c**) Local Manhattan plots (top) and LD heatmaps (bottom) at the candidate regions 108.745–111.067 Mb (Arahy.06), 136.002–139.998 Mb (Arahy.16), and 125.884–127.000 Mb (Arahy.18).

**Figure 8:**
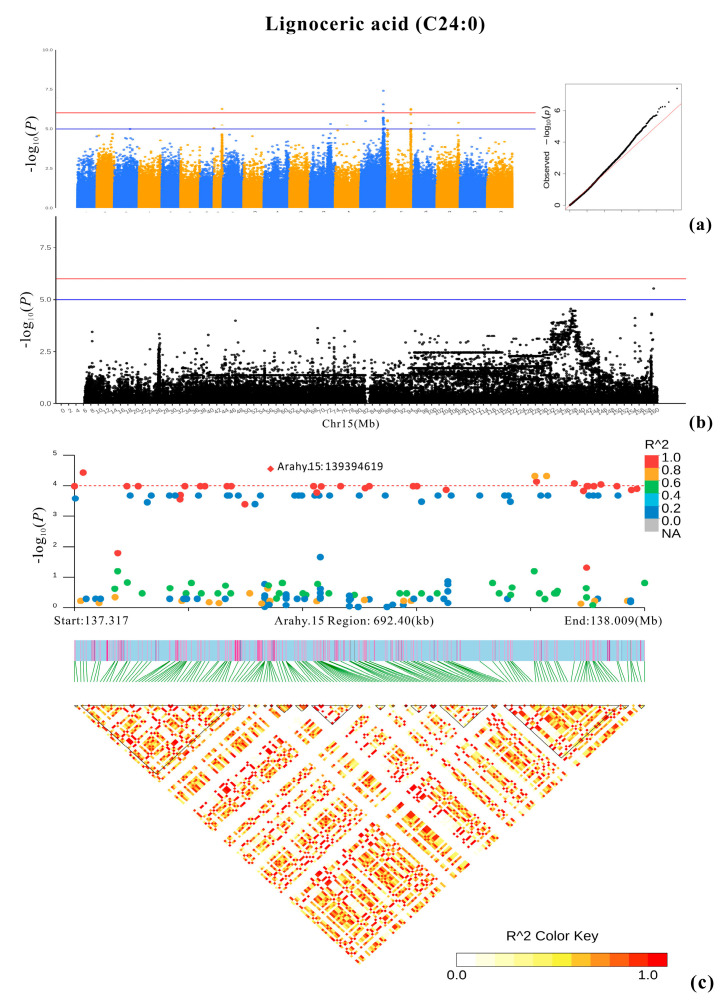
GWAS result for lignoceric acid. (**a**) Manhattan plot and QQ plot. The blue and red lines represent, respectively, the significance thresholds of −log_10_(*p*) = 5 and −log_10_(*p*) = 6. (**b**) Local Manhattan plots on Arahy.15. (**c**) Local Manhattan plots (top) and LD heatmaps (bottom) at regions 137.317–138.009 Mb (Arahy.15).

Both the TASSEL and EMMAX results were in agreement with each other. In total, 6-1195 SNPs were significantly associated with the nine studied fatty acids (Figure 4). The majority of those SNPs were detected for oleic acid (1195) and linoleic acid (1147), while arachidic acid had the third most significant SNPs (296) (Figure 5, Figure 6, Figure 7, Figure 8 and Figure S1; Table S2). All the other studied fatty acids had fewer than 100 significant SNPs. For oleic acid and linoleic acid, eight clear peak SNP clusters were identified (−log_10_(*p*) > 6) (Figure 5). Among these clusters, two peak SNP clusters on Arahy.09 (961 out of 1195) and Arahy.19 (919 out of 1147) contained the most significant SNPs associated with these two fatty acids (Table S2).

### 2.5. Co-Localized Candidate Regions

**Figure 9:**
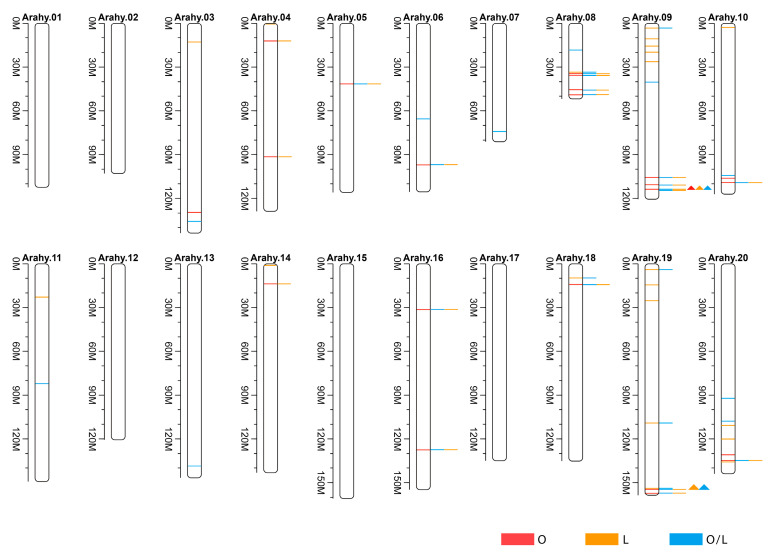
QTLs identified to be associated with oleic acid, linoleic acid, and the O/L ratio. The colorful lines represent the earlier identified QTLs, and the solid triangles point to the candidate regions that are identified by the present study. O: oleic acid; L: linoleic acid; O/L: the oleic/linoleic acid ratio.

Through a literature survey of early QTL mapping and GWAS studies of peanut oleic acid and linoleic acid, we found 22, 39, and 31 QTLs for oleic acid, linoleic acid, and the oleic/linoleic acid ratio, respectively (Table S3; Figure 9), which are widely distributed over 15 chromosomes. Among these QTLs, two regions on Arahy.09 (113.235–115.189 Mb) and Arahy.19 (155.091–155.200 Mb) overlap with the two most significant peak SNP clusters identified in the present study for oleic acid and linoleic acid. Gene annotations in these two regions revealed 348 candidate genes on Arahy.09 and 56 on Arahy.19 (Table S4), among which 226 on Arahy.09 and 2 on Arahy.19 were shared with the genes annotated in the two peak SNP clusters identified in the present study for oleic acid and linoleic acid (Table S5). Among those 226 shared candidate genes on Arahy.09, 3 are of special interest: *Arahy.42CZAS*, *Arahy.JYC97M,* and *Arahy.04JNDX*. *Arahy.04JNDX* is the closest gene to one of the most significant SNPs (Chr09: 114150503) associated with linoleic acid (−log_10_(*p*) = 16.86; Table 2), while the oleic/linoleic acid ratio differs dramatically between the two homozygotes at the SNPs located within *Arahy.42CZAS* (Chr09: 114195009) and *Arahy.JYC97M* (Chr09:114966251) according to the WGRS genotyping results (*p* values < 2.22 × 10^−16^; Figure S2; Table 2). 

Gene ontology (GO) analysis of those 226 shared candidate genes on Arahy.09 were mostly found in the cellular process, metabolic process, catalytic activity, and single-organism process. Kyoto Encyclopedia of Genes and Genomes (KEGG) pathway enrichment analysis showed that these 226 shared candidate genes were significantly enriched in five different pathways: protein processing in the endoplasmic reticulum, protein export, glycosaminoglycan degradation, other glycan degradation, and glycosphingolipid biosynthesis-ganglio series (Table S6).

### 2.6. Penta-Primer Amplification Refractory Mutation System (PARMS) Genotyping

**Figure 10:**
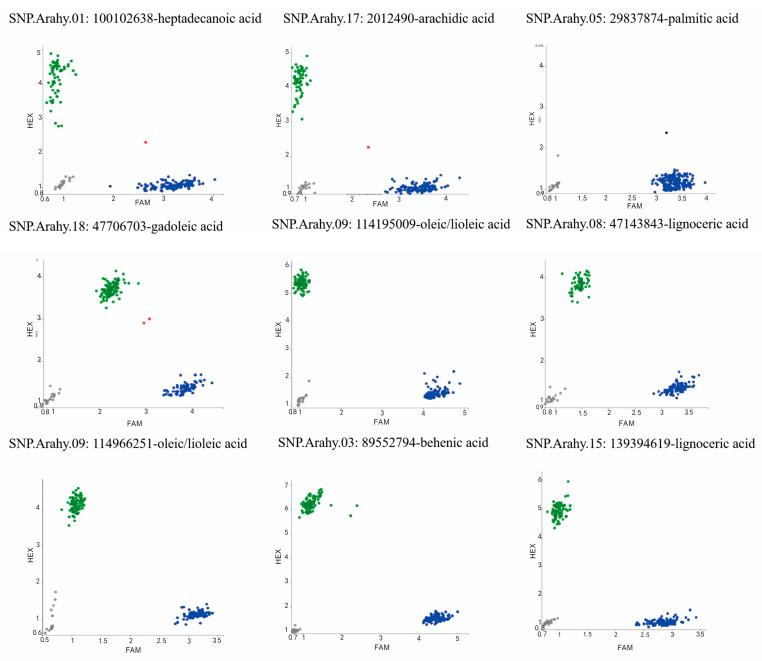
Penta-primer amplification refractory mutation system (PARMS) genotyping results. Green dots: HEX fluorescent signals; blue dots: FAM fluorescent signals; red dots: hybrid signals; gray dots: negative controls and inconclusive samples.

Nine selected SNPs that are associated with the studied fatty acids (except stearic acid) were genotyped using PARMS technology. The genotypes obtained with PARMS largely agree with those from whole-genome resequencing (WGRS) (*r* = 0.68–0.96, except for SNP_Chr05:29837874) (Table 3). Consistent with the GWAS results, the oleic/linoleic acid ratio differed significantly between genotypes “AA” and “GG” at SNP_Chr09:114966251 and between “GG” and “AA” at SNP_Chr09:114195009 (*p* value < 10^−37^) (the genotypes here were acquired using PARMS, Table 3; Figure 10). The lignoceric acid BLUP values were significantly different between genotypes “AA” and “GG” at Chr08:47143843 (*p* value < 10^−6^) and between genotypes “AA” and “GG” at SNP_Chr15:139394619 (*p* value < 10^−5^) (Table 3). Furthermore, the BLUP values of the heptadecanoic, gadoleic, and behenic acids significantly differed between the two homozygotes at SNP_Chr01:100102638 (*p* value < 10^−4^), SNP_Chr18:47706703 (*p* value = 0.007), and SNP_Chr03:89552794 (*p* value = 0.038) (Table 3), respectively. However, the arachidic acid BLUP values did not show a significant difference between genotypes “TT” and “CC” at SNP_Chr03:2012490, while only one allele was found with PARMS at SNP_Chr05: 29837874, which is associated with palmitic acid (Table 3).

### 2.7. qRT-PCR Verification

**Figure 11:**
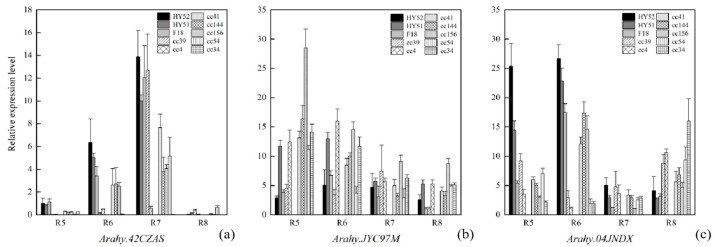
Comparison of the relative gene expression levels between the high- and low-oleic/linoleic-acid peanut accessions. The gene expression levels were obtained with qRT-PCR. High-oleic/linoleic-acid peanut accessions include cc4, cc39, HY52, HY51, and F18, while low-oleic/linoleic-acid accessions consist of cc54, cc34, cc41, cc144, and cc156. (**a**) *Arahy.42CZAS*; (**b**) *Arahy.JYC97M*; (**c**) *Arahy.04JNDX*.

The expression patterns of seven selected candidate genes were investigated with qRT-PCR at four different kernel developmental stages (R5-R8). Three of the selected candidate genes are the three abovementioned candidate genes on chromosome Arahy.09 that were detected by both earlier and the current studies for oleic acid and/or linoleic acid: *Arahy.42CZAS*, *Arahy.JYC97M,* and *Arahy.04JNDX*. The expression of *Arahy.42CZAS* (containing SNP_Chr09:114195009) increased gradually from R5 to R7. At R7, the expression of *Arahy.42CZAS* within the high-oleic peanut group was, on average, three times higher than that within the low-oleic peanut group (Figure 11). *Arahy.JYC97M* (containing SNP_Chr09:114966251 in its coding region; missense type) had the highest expression level at the kernel developmental stage R5 in the low-oleic acid accessions. In addition, *Arahy.04JNDX* is the nearest gene to SNP_Chr09:114150503, and it exhibits a higher expression level at R5 and R6 than at other stages (Figure 11).

**Figure 12:**
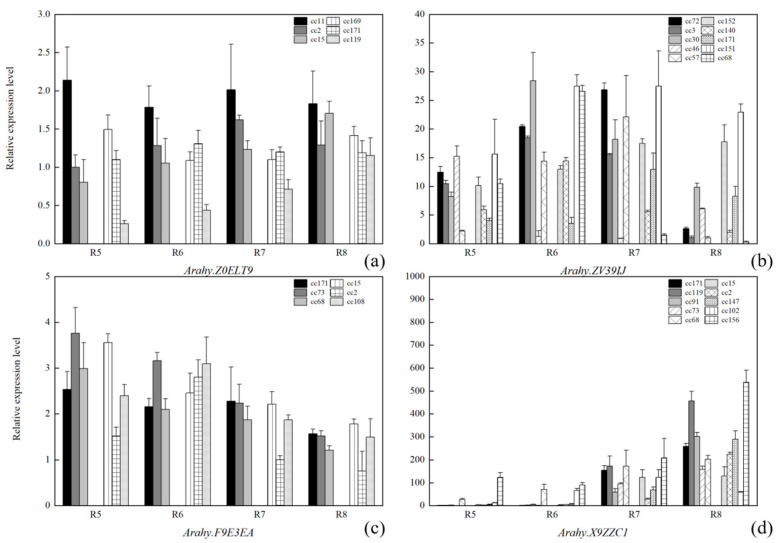
Comparison of the relative gene expression levels between the high- and low-palmitic/arachidic/gadoleic/lignoceric-acid peanut accessions. The gene expression levels were obtained with qRT-PCR. High-palmitic/arachidic/gadoleic/lignoceric-acid peanut accessions include, respectively, cc11, cc2, cc15/cc46, cc57, cc72, cc3, cc30/cc171, cc73, cc68/cc68, cc73, cc171, cc119, and cc91. Low-palmitic/arachidic/gadoleic/lignoceric-acid peanut accessions consist, respectively, of cc169, cc171, cc119/cc68, cc151, cc152, cc140, c171/cc2, cc15, cc108/cc2, cc15, cc102, cc156, and cc147. (**a**) *Arahy.Z0ELT9*; (**b**) *Arahy.ZV39IJ*; (**c**) *Arahy.F9E3EA*; (**d**) *Arahy.X9ZZC1*.

The remaining four selected genes involve significant SNPs for five of the other studied fatty acids. One of these selected genes is *Arahy.ZV39IJ*. It contains SNP_Chr17: 2012490, which is highly significantly associated with stearic acid (C18:0) and arachidic acid (C20:0) (−log_10_*P* = 6.93) (Figure S1; Table 2). This gene is much more highly expressed at developmental stages R6 and R7 than at R5 and R8 (Figure 12). The second selected gene, *Arahy.F9E3EA*, holds SNP_Chr18:47706703, which is a highly significant SNP associated with gadoleic acid (C20:1) (−log_10_*P* = 6.33) (Figure 7; Table 2). The expression level of *Arahy.F9E3EA* decreased from R5 to R8 (Figure 12). The third selected gene, *Arahy.X9ZZC1*, is the closest gene to SNP_Chr08: 47143843, which is one of the most significant SNPs associated with lignoceric acid (−log_10_*P* = 6.17, Table 2). *Arahy.X9ZZC1* exhibited a low level of expression at the kernel developmental stages R5 and R6 and a high level at R7 and R8. At R8, the high-lignoceric-acid group had higher expression levels of *Arahy.X9ZZC1* than the low-lignoceric-acid group (*p* value < 0.05) (Figure 12). However, the candidate gene (*Arahy.Z0ELT9*) for palmitic acid showed no significant differences among the four developmental stages, and there was no significant difference between the high- and low-palmitic-acid groups at each stage (Figure 12).

## 3. Discussion

The cultivated peanut is an important oilseed crop that is widely planted in the warm temperate, subtropical, and tropical zones between 35° N and 35° S [6,14]. In the present study, we analyzed 160 Chinese peanut landraces representing 82.4% of the genetic variation in Chinese landraces [15,16]. By combining our earlier acquired whole-genome resequencing (WGRS) data with fatty acid content data, we conducted a genome-wide association analysis (GWAS) and identified candidate genes responsible for the contents of nine fatty acids, including oleic and linoleic acids.

### 3.1. Known and Novel Candidate Genes Responsible for Oleic and Linoleic acid Content in Peanuts

Previous QTL/GWAS studies of peanut fatty acids mainly focused on oleic acid and linoleic acid. Many significant SNPs have been identified to be associated with these two major fatty acids, which are distributed on 15 peanut chromosomes, including Arahy.09 and Arahy.19 [6,9,10,12,13,17,18,19,20,21,22]. In the present study, most significant SNPs (>83%) for oleic acid and linoleic acid were detected within two peak SNP clusters on Arahy.09 and Arahy.19. These two SNP clusters overlap with the previously identified genomic regions for oleic and linoleic acids (Figure 5). Within the shared genomic regions on Arahy.09, the second most significant SNP_Chr9:114195009 for oleic acid is a known mutation for controlling the oleic/linoleic acid ratio in peanuts. This SNP is located within the gene *Arahy.42CZAS*, which codes for fatty acid desaturase 2, *FAD2A*. Within the *FAD2A* gene, the SNP_Chr09:114195009 is located at the first position of the amino acid codon for Asn150/Asp150 within the FAD2A gene, and the same A > G mutation was detected previously by Li et al. (2019) [23]. *FAD2* has been shown to be a key gene controlling the conversion of oleic acid to linoleic acid in the fatty acid synthesis process [24,25]. In addition to *Arahy.42CZAS*, our study also identified novel candidate genes that may affect oleic acid content, such as the *Arahy.04JNDX* gene. This gene is the nearest neighbor to the most significant SNP (Chr09:114150503) for linoleic acid and has been annotated as an MYB protein-coding gene. MYB proteins are a family of DNA-binding proteins that are particularly important in the transcriptional regulation of secondary metabolism and the cell cycle. One MYB family member, MYB76, has been shown to affect seed fatty acid accumulation by affecting fatty acid synthesis, modification, degradation, and oil body formation in *Arabidopsis* [26]. In addition, a mutation in another MYB family member, MYB89, was found to significantly promote the biosynthesis of major fatty acids in *Arabidopsis* seeds [27]. A second novel candidate gene that was discovered to be responsible for oleic acid and linoleic acid in this study is *Arahy.JYC97M*. Within its coding region, we found SNP_Chr09:114966251, which involves a missense mutation (A > G). The significant oleic/linoleic acid ratio difference between the genotypes at SNP_Chr09:114966251, as identified using GWAS, have been confirmed with PARMS analysis (Figure 10; Table 3). *Arahy.JYC97M* codes for a JmjC domain-containing protein; the histone demethylases of the JmjC domain regulate gene transcription by changing the methylation status of arginine (R) and lysine (Q) residues and play important roles in plant growth and development [13,28,29,30]. According to our qRT-PCR analysis, the expression of *Arahy.JYC97M* is the highest at an early kernel developmental stage (R5) within the low-oleic-acid peanut accessions (AA), suggesting that this early overexpression of *Arahy.JYC97M* is likely to have activated the epigenetic regulation, which may eventually contribute to the low oleic acid content [31,32,33]. However, peanut oleic and linoleic acid contents are complex quantitative traits controlled by multiple genes and influenced by the environment [34]. Therefore, the performance of different peanut accessions during peanut breeding needs to consider not only genotypes but also environments and genotype-and-environment interactions.

### 3.2. Candidate Genes for the Other Fatty Acids

The estimated broad-sense heritabilities (0.74 < H^2^ < 0.85) of the other seven studied fatty acids are high, indicating that the variation in these peanut fatty acids is primarily attributed to genetic factors. However, compared with oleic acid and linoleic acid, fewer QTL mapping/GWAS studies have explored the genetic basis of these fatty acids thus far [6,35]. In the current study, we have identified novel candidate genes for those fatty acids. For example, *Arahy.ZV39IJ* holds the highly significant SNP for stearic acid (C18:0) and arachidic acid (C20:0). This gene codes for an oligopeptide transporter that plays a role in the transmembrane transport of plant secondary metabolites, metabolites, hormones, and other substances [36].

The candidate gene for gadoleic acid (C20:1), *Arahy.F9E3EA*, is the closest gene to a highly significant SNP (Chr18:47706703) for this acid. *Arahy.F9E3EA* encodes an F-box/LRR protein. F-box/LRR proteins have been shown to be involved in plant growth and development, senescence, biological/abiotic stress responses, and other biological processes [37,38].

For lignoceric acid (C24:0), the identified candidate gene, *Arahy.X9ZZC1*, is the closest gene to the second most significant SNP (Chr08:47143843) for this acid. *Arahy.X9ZZC1* codes for ethylene-responsive transcription factor (ERF) 3-like in Glycine max. ERF belongs to the AP2/ERF superfamily in plants [39], and the WRI transcription factors of the AP2/ERF superfamily have been shown to play important roles in the synthesis of fatty acids [40]. For instance, WRI4 can upregulate LACS1 (long-chain acyl-CoA synthetase 1) to participate in the synthesis of long-chain fatty acids [41]. The candidate gene for lignoceric acid, *Arahy.U6RNCV*, is the closest gene to the most significant SNP (Chr15:139394619) for this acid. *Arahy.U6RNCV* codes for dihydropyrimidine dehydrogenase (DPD), which is primarily involved in pyrimidine metabolism and plays a similar role in metabolizing 5-FU, a pyrimidine analog [42].

## 4. Materials and Methods

### 4.1. Plant Materials and Phenotype Collection

A total of 160 key peanut germplasms were cultivated at three locations (Dongying, Heze, and Laixi) in China from 2020 to 2021 (Table S7). Thirty to thirty-four individuals from each accession were planted in a two-row plot (3.00 m long and 0.80 m wide). After the harvest, the fatty acid composition of these peanut accessions was determined using gas chromatography-mass spectrometry (GC-MS). For each accession, 8–10 dry seeds were ground and sifted through a 20-mesh sieve (Shangyu Hujiang Instrument Factory, Zhejiang, China). We added 0.2 g of the acquired seed powder to a reaction mix containing a 2 mL mixture of diethyl ether and petroleum ether (1:1) (Aladdin, Shanghai, China). The reaction mix was allowed to stand for 30 min before adding 1 mL 0.4 mol/L potassium hydroxide-methanol solution (Kermel, Tianjin, China). After vortexing the reaction mix, it was left to stand for 1 h. Following this, 2 mL ultrapure water was added while ensuring the supernatant remained clear (>30 min). Finally, the reaction mix was diluted 1000 times with petroleum ether (temperature range: 60–90 °C) (Kermel, Tianjin, China).

Fatty acid composition was determined using Agilent 7890A gas chromatography (Agilent Technologies, Santa Clara, CA, USA) with an HP-88 capillary column (130 m × 0.25 mm × 0.20 μm). The carrier gas used was helium, and the column was initially set at 210 °C for 9 min, with a heating rate of 20 °C/min. The temperature was then programmed to 230 °C and maintained at this temperature for 8 min. The shunt ratio was set at 30:1, and the detector temperature was 300 °C. The hydrogen flow rate was 40 mL/min, the air flow rate was 400 mL/min, and the high-purity helium flow rate was 10 mL/min. The peak area and percentage of fatty acid composition were determined using an Agilent integrator. The fatty acid was determined by comparing the retention time with the fatty acid methyl ester standard (Sigma-Aldrich, Shanghai, China). The relative proportion of total peak area was utilized to determine the fatty acid contents. To minimize environmental effects, BLUP (best linear unbiased prediction) values were estimated for each fatty acid and used in subsequent GWAS analyses [43]. The correlation coefficient between each pair of the analyzed traits was calculated with the R function “cor” (https://cran.r-project.org/bin/windows/base/, accessed on 10 March 2021), and the broad-sense heritability (H^2^) of each trait was estimated using the R package “lem4”.

### 4.2. High-Density SNP Identification from Whole-Genome Resequencing (WGRS) Data

Whole-genome resequencing data are publicly available for the 160 studied peanut accessions in the Sequence Read Archive (SRA) database with the accession number PRJNA857148. This dataset was downloaded, and the high-quality reads from the dataset were aligned to a cultivated peanut reference genome (*Arachis hypogaea* cv. Tifrunner v1; https://www.peanutbase.org) using BWA v0.7.15, allowing < 4% mismatch and maximum one gap. GATK’s Unified Genotyper v4.0 was used to identify SNPs (https://software.broadinstitute.org/gatk). SNP filtering used the following criteria: (1) call quality divided by depth (QD) > 2.0; (2) mapping quality (MQ) > 40.0; (3) missing genotype rate (MGR) < 20%; (4) minor allele frequency (MAF) > 0.05; (5) Fisher’s exact test (FS) > 60.0; (6) cluster window size equal to 5 and cluster size equal to 2.

### 4.3. Genome-Wide Association Study (GWAS)

To identify genomic loci that are significantly associated with the studied fatty acid traits, GWAS analysis was performed using TASSEL v5.2.3 and EMMAX (efficient mixed model association expedited) [44,45]. A mixed linear model (MLM; Y = Xa + Qb + Ku + e, where Y denotes the phenotype, X stands for the genotypes at each SNP locus, Q represents population structure, K is the relationship between samples, and e stands for residual error) was selected for each trait. A matrix of pairwise kinship coefficients was calculated using SPAGeDi v1.5 [46]. The *p*-value threshold for significant associations was set to 10–6. The triangular correlation heatmap was generated with LDBlockShow v2.6.3 [47]. Both GO (gene ontology) enrichment analysis and KEGG (Kyoto Encyclopedia of Genes and Genomes pathway database) pathway enrichment analysis were conducted using the omicshare web server (www.omicshare.com/tools).

### 4.4. Literature Survey of Early QTL/GWAS Studies on Peanut Oleic and Linoleic Acids

To narrow down the candidate SNP list, we conducted a literature survey and compared our significant SNPs with QTLs detected in early QTL/GWAS studies of peanut oleic and linoleic acids [6,9,10,12,13,17,18,19,20,21,22]. The genomic regions for those earlier identified QTLs were determined using the left/right marker sequences that are publicly available (Table S3).

### 4.5. PARMS Genotyping

To validate the GWAS results, genotyping of nine selected SNPs (Table S8) on 160 samples was carried out with the penta-primer amplification refractory mutation system (PARMS) (Gentides, Wuhan, China) [48]. Primers were designed with Primer Premier 5.0 (Table S3). After DNA extraction from each sample, PCR reactions were set in 160-well PCR plates for PARMS genotyping. Each PCR reaction well (5 μL) contained 2× PARMS PCR reaction mix, allele-specific primers (150 nM each), 400 nM locus-specific primer, and 1.4 μL of DNA template. Five microliters of mineral oil were then added into each reaction well to prevent evaporation. The thermal cycler program for PARMS started with a 15 min denaturation step at 95 °C. This was followed by 10 cycles of denaturation (95 °C for 20 s) and annealing (1 min, started at 65 °C, and then decreased 0.8 °C per cycle until reaching 57 °C). Subsequently, there were 32 cycles of denaturation at 95 °C for 20 s and annealing at 57 °C for 1 min. The 160-well PCR plates were read using a TECAN infinite M1000 plate reader. SNP calling was carried out with an online software SNPdecoder (http://www.snpway.com/snpdecoder/) combined with manual modification. In each genotyping, three main types of samples may be found: samples with homozygotes (majority), samples with heterozygotes, and samples with negative or inconclusive genotypes. The significance of the phenotypic differences between the genotypes at each SNP were detected using a Student’s *t*-test.

### 4.6. qRT-PCR Verification

To investigate the expression patterns of the top SNPs, three to five high-content peanut accessions and three to five low-content accessions for each of the six of the studied fatty acids were planted in Qingdao in 2021 (Table S8). Among those peanut accessions, we included three extra-high oleic acid improved varieties (>75%), HY51, HY52, and F18, which were not included in the 160 samples for GWAS analysis. The seeds were collected from four kernel developmental stages that correspond to the R5-R8 stages as defined by Boote (1982) [49]. Total RNA was extracted using the EASY spin plant RNA kit (Ailab, Beijing, China). Subsequently, all samples were treated with DNase I (Takara, Shanghai, China), and the concentration of RNA was determined using a NanoDrop^®^ ND-1000 (Thermo, Shanghai, China). Next, the obtained RNA was reverse transcribed into cDNA using M-MLV reverse transcriptase, and qRT-PCR for seven candidate genes was performed with the BYBR Premix Ex Taq Kit (Takara, Osaka, Japan) on a Step One system (Applied Biosystems, Carlsbad, CA, USA) (Table S9). The qRT-PCR reaction consists of an initial denaturation step at 95 °C for 10 min, followed by 40 cycles of 95 °C for 15 s and 60 °C for 30 s. The relative expression levels of each gene were calculated using the 2^−∆∆Ct^ method that normalized gene expression to a reference gene (Actin) with three biological replicates.

## 5. Conclusions

The peanut is a globally significant oilseed crop, and its oil quality is primarily determined by its fatty acid composition. Here in the current study, we conducted GWAS analysis of nine fatty acids, including oleic acid and linoleic acid, using the available whole-genome resequencing data. For oleic and linoleic acids, the two most significant peak SNP clusters (on Arahy.09 and Arahy.19) were found to overlap with previously identified QTLs that are responsible for oleic and linoleic acid contents. Among the candidate genes annotated from the overlapping regions, we identified both known (*FAD2*) and novel candidate genes. In addition, we identified candidate genes for other important fatty acids. Additionally, we also identified candidate genes for other important fatty acids. However, it is worth noting that a significant SNP does not always indicate a functional difference, and the polymorphisms or functional genes in proximity need to undergo functionality testing. Nevertheless, our results may hold great potential for future peanut oil quality improvement.

## Figures and Tables

**Table 1:** Phenotypic statistics of nine peanut fatty acids under three environmental conditions.

Traits	Environment	Maximum	Minimum	Mean	SD	CV	H2
Palmitic acid (%)	LX	19.67	11.81	16.17	1.35	5.53	0.84
	DY	18.74	11.26	15.63	1.2		
	HZ	18.81	10.94	14.52	1.71		
	BLUP	18.38	13.13	15.46	0.84		
Stearic acid (%)	LX	6.71	2.18	4.52	0.86	7.96	0.85
	DY	6.79	1.95	4.39	0.83		
	HZ	6.47	1.98	4.45	0.85		
	BLUP	5.91	2.98	4.48	0.35		
Oleic acid (%)	LX	52.02	28.89	38.97	5.36	5.22	0.92
	DY	47.32	28.76	36.18	4.61		
	HZ	49.58	29.75	38.85	5.62		
	BLUP	48.13	30.8	37.97	2		
Linoleic acid (%)	LX	42.48	23.06	34.65	4.38	5.24	0.92
	DY	45.14	30.29	37.84	3.83		
	HZ	45.84	26.09	36.52	5.05		
	BLUP	43.03	28.53	36.35	1.87		
Arachidic acid (%)	LX	2.42	1.07	1.74	0.25	5.69	0.84
	DY	2.38	1.06	1.72	0.23		
	HZ	2.36	1.06	1.76	0.23		
	BLUP	2.24	1.2	1.74	0.1		
Arachidonic acid (%)	LX	1.45	0.2	0.61	0.2	10.05	0.85
	DY	1.33	0.41	0.67	0.19		
	HZ	1.14	0.31	0.62	0.17		
	BLUP	1.19	0.41	0.63	0.06		
Behenic acid (%)	LX	3.68	1.26	2.64	0.46	8.03	0.79
	DY	4.28	1.58	2.64	0.53		
	HZ	3.78	1.36	2.49	0.46		
	BLUP	3.4	1.77	2.58	0.21		
Lignoceric acid (%)	LX	1.09	0.2	0.59	0.17	13.06	0.74
	DY	1.2	0.25	0.69	0.2		
	HZ	1.13	0.31	0.65	0.15		
	BLUP	0.89	0.43	0.65	0.09		
Heptadecanoic acid (%)	LX	0.16	0.00	0.06	0.04	4.65	0.71
	DY	0.28	0.01	0.10	0.04		
	HZ	0.13	0.00	0.06	0.03		
	BLUP	0.02	−0.01	0.000008	0.004		

NOTE: SD, standard deviation. CV, coefficient of variance. H^2^, broad-sense heritability.

**Table 2:** The most significant SNPs and the corresponding candidate genes for different fatty acids in peanuts.

Traits	Formula	Chromosome	SNP Marker	−log_10_*P*	Candidate Gene	Regions
Palmitic acid	C16:0	05	29837874	6.01	Z0ELT9_29894459_29894933; 94M007_29703175_29707896	intergenic_region; intergenic_region
Heptadecanoic acid	C17:0	13	76312411	7.11	19YNSD_75079855_75095251; 4AY3MY_76555084_76557170	intergenic_region; intergenic_region
Oleic acid	C18:1	09	114431906	18.91	Q6VS78_114441903_114454826; EZ44CQ_114398207_114402961	intergenic_region; intergenic_region
Oleic acid	C18:1	09	114195009	16.51	42CZAS_114192195_114195899; NEG1KJ_114197025_114199514	missense_variant; upstream_gene_variant
Oleic acid	C18:1	09	114966251	11.53	JYC97M_114964545_114971892	missense_variant
Oleic acid	C18:1	09	113238925	11.36	W8BHQ1_113237385_113243056	missense_variant
Oleic acid	C18:1	19	155180119	13.01	X7PJ8H_155166355_155175712; 27N6DD_155178523_155181610	upstream_gene_variant; missense_variant
Oleic acid	C18:1	19	155129095	12.52	MZJT69_155128975_155136731; 96PB6J_155123874_155125079	missense_variant; upstream_gene_variant
Oleic acid	C18:1	19	155194719	11.81	M9I28E_155193126_155195795	missense_variant
Oleic acid	C18:1	19	155097696	7.86	HNK57V_155093492_155094611; H41NY2_155096003_155098040; 2TIK4C_155088309_155093083	upstream_gene_variant; missense_variant; upstream_gene_variant
Linoleic acid	C18:2	09	114150503	16.86	04JNDX_114163849_114165495; HL6BNW_114136486_114141437	intergenic_region; intergenic_region
Linoleic acid	C18:2	09	114038775	16.52	BKP6F9_114036941_114040904; MQG1NS_114028309_114036421; DNVP1U_114042573_114046728	intron_variant; downstream_gene_variant; downstream_gene_variant
Stearic acid/arachidic acid	C18:0/C20:0	17	2012490	6.93	ZV39IJ_2005402_2013748	intron_variant
Stearic acid/arachidic acid	C18:0/C20:0	14	93225212	6.57	1D7P49_4778370_4784309	upstream_gene_variant
Gadoleic acid	C20:1	18	47706703	6.33	BHV928_48473410_48481907; F9E3EA_47603978_47613948	intergenic_region; intergenic_region
Behenic acid	C22:0	02	11398245	7.55	RVN5Z1_11401650_11402727	upstream_gene_variant
Behenic acid	C22:0	03	89552794	5.12	U2P2B1_89414239_89414749; RT27P1_89571644_89576434	intergenic_region; intergenic_region
Lignoceric acid	C24:0	08	47143843	6.17	X9ZZC1_47160111_47162179; CJP1WL_47130716_47133118	intergenic_region; intergenic_region
Lignoceric acid	C24:0	15	139394619	6.51	U6RNCV_139428034_139433066; 4KZ99U_139272115_139273282	intergenic_region; intergenic_region

**Table 3:** Information table for the nine selected SNPs that were validated using the penta-primer amplification refractory mutation system (PARMS) analysis. Columns 5 and 6 are for the WGRS genotyping result, while columns 7–9 are for the PARMS genotyping results.

Chr.	SNP Position	Associated Traits	WGRS Genotypes	Genotypes No.	PARMS Genotype	Fluorescent Labels	*p*-Values (Phenotypic Differences between Genotypes)	Correlation (*r*) between WGRS and PARMS Genotyping
09	114195009	oleic acid (C18:1)/linoleic acid (C18:2)	Absent; AG; AA; GG	13; 1; 69; 75	AA/GG	FAM(76H)/HEX(83L)	3.58586 × 10^−46^/ 1.01058 × 10^−38^	0.942617367
09	114966251	oleic acid (C18:1)/linoleic acid (C18:2)	Absent; GA; AA; GG	10; 1; 73; 74	GG/AA	FAM (82L)/HEX(77H)	4.58 × 10^−38^	0.812028955
05	29837874	palmitic acid (C16:0)	Absent; GA; AA; GG	0; 1; 66; 91	GG/AA	FAM	--	2.52572E-34
01	100102638	heptadecanoic acid (C17:0)	Absent; CT; TC; TT; CC	0; 1; 3; 56; 98	CC/TT	FAM (101L)/HEX (57H)	8.65 × 10^−5^	0.958436351
17	2012490	arachidic acid (C20:0)	Absent; TC; TT; CC	0; 2; 55; 101	CC/TT	FAM (72H)/HEX (85L)	0.469692082	0.900815724
18	47706703	gadoleic acid (C20:1)	Absent; AG; GA; GG; AA	0; 1; 1; 67; 89	GG/AA	FAM (72H)/HEX (85L)	0.007222919	0.767613482
03	89552794	behenic acid (C22:0)	Absent; TT; CC	0; 75; 83	CC/TT	FAM (83H)/HEX(76L)	0.038232227	0.718998755
08	47143843	lignoceric acid (C24:0)	Absent; GA; GG; AA	0; 1; 60; 97	AA/GG	FAM (97H)/HEX(62L)	4.10 × 10^−7^	0.941170491
15	139394619	lignoceric acid (C24:0)	Absent; GG; AA	0; 63; 95	AA/GG	FAM(94H)/HEX(65L)	7.00 × 10^−6^	0.680423542

## Data Availability

Data are contained within the article and Supplementary Materials.

## Supplementary Materials

The following are available online at https://www.mdpi.com/article/10.3390/plants13010016/s1. Figure S1: Manhattan plots showing SNPs associated with nine different fatty acids and the oleic/linoleic ratio. For each trait, the Q-Q plots are also displayed on the right. The blue and red horizontal lines represent, respectively, the significance thresholds of −log10 (p) = 5 and/or −log10 (p) = 6. (N: Northern latitudes; E: Eastern longitude). Figure S2: Phenotypic comparison between the genotypes at each SNP based on genome-wide association study (GWAS) results. Table S1: SNP and Indel distributions on each of the 20 peanut chromosomes. Table S2: The number of significant SNPs that are identified using GWAS for each of the 9 studied fatty acids on each of the 20 peanut chromosomes. Table S3: Information table for the 90 QTLs that were identified by earlier studies. These QTLs were found to be associated with oleic acid, linoleic acid, and the oleic/linoleic ratio. Table S4: Information table for the 404 candidate genes annotated from 2 genomic regions on Arahy.09 (113.2–115.2 Mb) and Arahy.19 (155.0–155.2 Mb) that were identified with available QTL mapping and GWAS studies of peanut oleic acid and linoleic acid. Table S5: Information table for the 226 shared candidate genes annotated on Arahy.09. These 226 genes are shared between early studies and the current study. Table S6: Enriched GO pathways and KEGG pathway among the 226 candidate genes identified on Arahy.09 that are shared between early studies and the current study. Table S7: Information table for the 160 studied peanut accessions. Table S8: Primers used in the penta-primer amplification refractory mutation system (PARMS) analysis. Table S9: Information table for the 226 shared candidate genes annotated on Arahy.09. These 226 genes are shared between early studies and the current study. Table S10: High-content peanut accessions and low-content accessions for each of the six fatty acids used in qRT-PCR.

## References

[1] Rachaputi R.C.N., Wright G. (2016). The World of Food Grains. Encyclopedia of Food Grains.

[2] O’Byrne D.J., Knauft D.A., Shireman R.B. (1997). Low fat monounsaturated rich diets containing high-oleic peanuts improve serum lipoprotein profiles. Lipids.

[3] Yamaki T., Nagamine I., Fukumoto K., Yano T., Miyahara M., Sakurai H. (2005). High oleic peanut oil modulates promotion stage in lung tumorigenesis of mice treated with methyl nitrosourea. Food Sci. Technol. Res..

[4] Chibisa G.E., Gorka P., Penner G.B., Berthiaume R., Mutsvangwa T. (2015). Effects of partial replacement of dietary starch from barley or corn with lactose on ruminal function, short-chain fatty acid absorption, nitrogen utilization, and production performance of dairy cows. J. Dairy Sci..

[5] Harwood J.L. (2005). Fatty acid biosynthesis. Plant Lipids: Biology, Utilization and Manipulation.

[6] Otyama P.I., Kulkarni R., Chamberlin K., Ozias-Akins P., Chu Y., Lincoln L.M., MacDonald G.E., Anglin N.L., Dash S., Bertioli D.J. (2020). Genotypic characterization of the us peanut core collection. G3 Genes Genom. Genet..

[7] Dar T.U.H., Rehman R.U. (2017). Polyploidy: Recent trends and Future Perspectives.

[8] Okuley J., Lightner J., Feldmann K., Yadav N., Lark E., Browse J. (1994). *Arabidopsis FAD2* gene encodes the enzyme that is essential for polyunsaturated lipid synthesis. Plant Cell.

[9] Zhao Q., Wu J., Cai G., Yang Q., Shahid M., Fan C., Zhang C., Zhou Y. (2019). A novel quantitative trait locus on chromosome A9 controlling oleic acid content in *Brassica napus*. Plant Biotechnol. J..

[10] Pandey M.K., Wang M.L., Qiao L.X., Feng S.P., Khera P., Wang H., Tonnis B., Barkley J.P., Wang N.A., Holbrook C.C. (2014). Identification of QTLs associated with oil content and mapping *FAD2* genes and their relative contribution to oil quality in peanut (*Arachis hypogaea* L.). BMC Genet..

[11] Hake A.A., Shirasawa K., Yadawad A., Sukruth M., Patil M., Nayak S.N., Lingaraju S., Patil P.V., Nadaf H.L., Gowda M.V.C. (2017). Mapping of important taxonomic and productivity traits using genic and non-genic transposable element markers in peanut (*Arachis hypogaea* L.). PLoS ONE.

[12] Hu X.H., Zhang S.Z., Miao H.R., Cui F.G., Shen Y., Yang W.Q., Xu T.T., Chen N., Chi X.Y., Zhang Z.M. (2018). High-density genetic map construction and identification of QTLs controlling oleic and linoleic acid in Peanut using SLAF-seq and SSRs. Sci. Rep..

[13] Zhang H., Wang M.L., Dang P., Jiang T., Zhao S.Z., Lamb M., Chen C. (2021). Identification of potential QTLs and genes associated with seed composition traits in peanut (*Arachis hypogaea* L.) using GWAS and RNA-Seq analysis. Gene.

[14] Yu S.L. (2011). Peanut Genetics and Breeding in China.

[15] Yan C.X., Li C.J., Zheng Y.X., Han Z.Q., Chen J., Wang J., Shan S.H. (2019). Screening key germplasms from Chinese peanut landraces. Shandong Agric. Sci..

[16] Yan C.X., Wang J., Zhang H., Li C.J., Song X.X., Sun Q.X., Yuan C.L., Zhao X.B., Shan S.H. (2020). Developing the key germplasm of Chinese peanut landraces based on phenotypic traits. Acta Agron. Sin..

[17] Sarvamangala C., Gowda M., Varshney R.K. (2011). Identification of quantitative trait loci for protein content, oil content and oil quality for groundnut (*Arachis hypogaea* L.). Field Crops Res..

[18] Shasidhar Y., Vishwakarma M.K., Pandey M.K., Janila P., Variath M.T., Manohar S.S., Nigam S.N., Guo B., Varshney R.K. (2017). Molecular mapping of oil content and fatty acids using dense genetic maps in groundnut (*Arachis hypogaea* L.). Front. Plant Sci..

[19] Liu H. (2011). Inheritance of Main Traits Related to Yield and Quality, and Their QTL Mapping in Peanut (*Arachis hypogaea* L.). Master’s Thesis.

[20] Zhang X.Y., Han S.Y., Xu J., Yan M., Liu H., Tang F.S., Dong W.Z., Huang B.Y. (2012). Identification of QTLs for important quality traits in cultivated peanut (*Arachis hypogaea* L.). Chin. J. Oil Crop Sci..

[21] Li X.P., Xu X.J., Cai Y., Guo J.B., Huang L., Ren X.P., Li Z.D., Chen W.G., Luo H.Y., Zhou X.J. (2016). Quantitative trait locus analysis for main quality traits in cultivated peanut (*Arachis hypogaea* L.). Chin. J. Oil Crop Sci..

[22] Zhang X.G., Zhang J.H., He X.Y., Wang Y., Ma X.L., Yin D.M. (2017). Genome-wide association study of major agronomic traits related to domestication in Peanut. Plant Sci..

[23] Li L., Cui S.L., Mu G.J., Yang X.L., Hou M.Y., Li W.P., Liu F.Q., Liu L.F. (2019). Research progress of peanut breeding with high oleic acid. Chin. J. Oil Crop Sci..

[24] Ruan J., Shan L., Li X.G., Guo F., Meng J.J., Wan S.B., Peng Z.Y. (2018). Genome-wide identification and expression pattern analysis of peanut FAD gene family. Shandong Agric. Sci..

[25] Wang Y., Zhang X.G., Zhao Y.L., Prakash C.S., He G.H., Yin D.M. (2015). Insights into the novel members of the FAD2 gene family involved in high-oleate fluxes in peanut. Genome.

[26] Duan S.W., Jin C.Y., Li D., Gao C.H., Qi S.H., Liu K.G., Hai J.B., Ma H.L., Chen M.X. (2017). MYB76 Inhibits seed fatty acid accumulation in *Arabidopsis*. Front. Plant Sci..

[27] Li D., Jin C.Y., Duan S.W., Zhu Y.N., Qi S.H., Liu K.G., Gao C.H., Ma H.L., Zhang M., Liao Y.H. (2017). MYB89 transcription factor represses seed oil accumulation. Plant Physiol..

[28] Jung S., Powell G., Moore K., Abbott A. (2000). The high oleate trait in the cultivated peanut (*Arachis hypogaea* L.). II. Molecular basis and genetics of the trait. Mol. Gen. Genet..

[29] Chu Y., Holbrook C.C., Peggy Q.A. (2009). Two alleles of *ah FAD2B* control the high oleic acid trait in cultivated peanut. Crop Sci..

[30] Zhang J. (2021). Identification and Phylogenetic Analysis of the JmjC Domain-Containing Histone Demethylase Gene Family in Plants. Master of Thesis.

[31] Wan Y.S., Li X.D., Fan H. (1995). Relationship between arachidonic acid/linoleic acid ratio and sowing time and temperature. Shandong Agric. Sci..

[32] Klose R.J., Kallin E.M., Zhang Y. (2006). JmjC-domain-containing proteins and histone demethylation. Nat. Rev. Genet..

[33] Han Y., Li X., Cheng L., Liu Y., Wang H., Ke D., Yuan H., Zhang L., Wang L. (2016). Genome-wide analysis of soybean JmjC domain-containing proteins suggests evolutionary conservation following whole-genome duplication. Front. Plant Sci..

[34] Zhang H., Yu Y., Wang M., Dang P., Chen C. (2023). Effect of genotype-by-environment interaction on oil and oleic fatty acid contents of cultivated peanuts. Horticulturae.

[35] Wu B., Liu N., Huang L., Luo H.Y., Zhou X.J., Chen W.G., Guo J.B., Huai D.X., Xia Y.F., Lei Y. (2022). Identification of markers stably associated with different fatty acid content in peanut through association analysis. J. Oil Crop Sci..

[36] Wang C.H., Wang X.Q., Li J.X., Guan J.H., Tan Z.J., Zhang Z., Shi G.R. (2022). Genome-wide identification and transcript analysis reveal potential roles of oligopeptide transporter genes in iron deficiency induced cadmium accumulation in peanut. Front. Plant Sci..

[37] Fang Q., Zhou F.L., Zhang Y., Singh S., Huang C.F. (2021). Degradation of STOP1 mediated by the F-box proteins RAH1 and RAE1 balances aluminum resistance and plant growth in *Arabidopsis thaliana*. Plant J..

[38] Xu K.H., Zhao Y., Zhao Y., Feng C., Zhang Y.H., Wang F.W., Li X.W., Gao H.T., Liu W.C., Jing Y. (2022). Soybean F-box-like protein GmFBL144 interacts with small heat shock protein and negatively regulates plant drought stress tolerance. Front. Plant Sci..

[39] Min T., Liu C.E., Xie J., Yi Y., Wang L.M., Ai Y.W., Wang H.X. (2019). Effects of vacuum packaging on enzymatic browning and ethylene response factor (ERF) gene expression of fresh-cut lotus root. HortScience.

[40] Xu S.D., Geng X.M., Wang L.L. (2021). A review of the structure, function and expression regulation of ethylene response factors (ERF) in plant. J. Zhejiang AF Univ..

[41] Gao Y., Yang Y.L., Li M.Z., He L.X., Li H. (2021). Cloning, bioinformatic analysis and expression vector construction of broccoli WRI4 gene. Jiangsu Agric. Sci..

[42] Sharma V., Gupta S.K., Verma M. (2019). Dihydropyrimidine dehydrogenase in the metabolism of the anticancer drugs. Cancer Chemother. Pharm..

[43] Davey J.W., Hohenlohe P.A., Etter P.D., Boone J.Q., Catchen J.M., Blaxter M.L. (2011). Genome-wide genetic marker discovery and genotyping using next-generation sequencing. Nat. Rev. Genet..

[44] Bradbury P.J., Zhang Z., Kroon D.E., Casstevens T.M., Ramdoss Y., Buckler E.S. (2007). Tassel: Software for association mapping of complex traits in diverse samples. Bioinformatics.

[45] Kang H.M., Sul J.H., Service S.K., Zaitlen N.A., Kong S.Y., Freimer N.B., Sabatti C., Eskin E. (2010). Variance component model to account for sample structure in genome-wide association studies. Nat. Genet..

[46] Hardy O.J., Vekemans X. (2002). Spagedi: A versatile computer program to analyse spatial genetic structure at the individual or population levels. Mol. Ecol. Notes.

[47] Dong S.S., He W.M., Ji J.J., Zhang C., Guo Y., Yang T.L. (2021). LDBlockShow: A fast and convenient tool for visualizing linkage disequilibrium and haplotype blocks based on variant call format files. Brief. Bioinform..

[48] Lu J., Hou J., Ouyang Y., Luo H., Zhao J.H., Mao C., Han M., Wang L., Xiao J.H., Yang Y.Y. (2020). A direct PCR-based SNP marker–assisted selection system (D-MAS) for different crops. Mol. Breed..

[49] Boote K.J. (1982). Growth stages of peanut (*Arachis hypogaea* L.). Peanut Sci..

