# Supplementary figures and images for: Identification of Quantitative Trait Nucleotides and Development of Diagnostic Markers for Nine Fatty Acids in the Peanut
Source: Plants (Basel). 2023 Dec 20;13(1):16. doi: 10.3390/plants13010016 (PMC10780752; doi:10.3390/plants13010016)

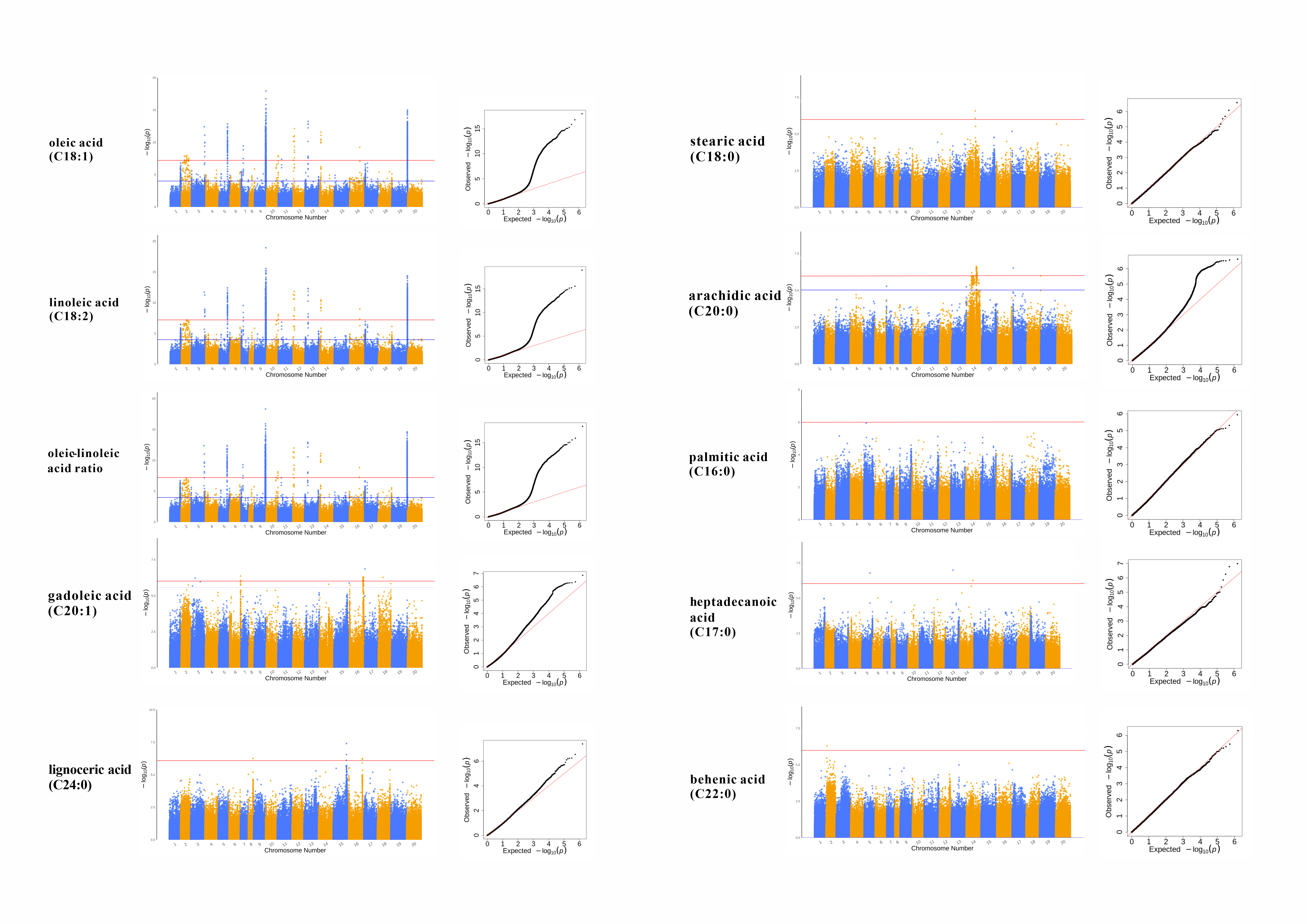

Supplement: Supplementary file 1 [file plants-13-00016-s001.zip › plants-2749434-supplementary/Figure S1.png]

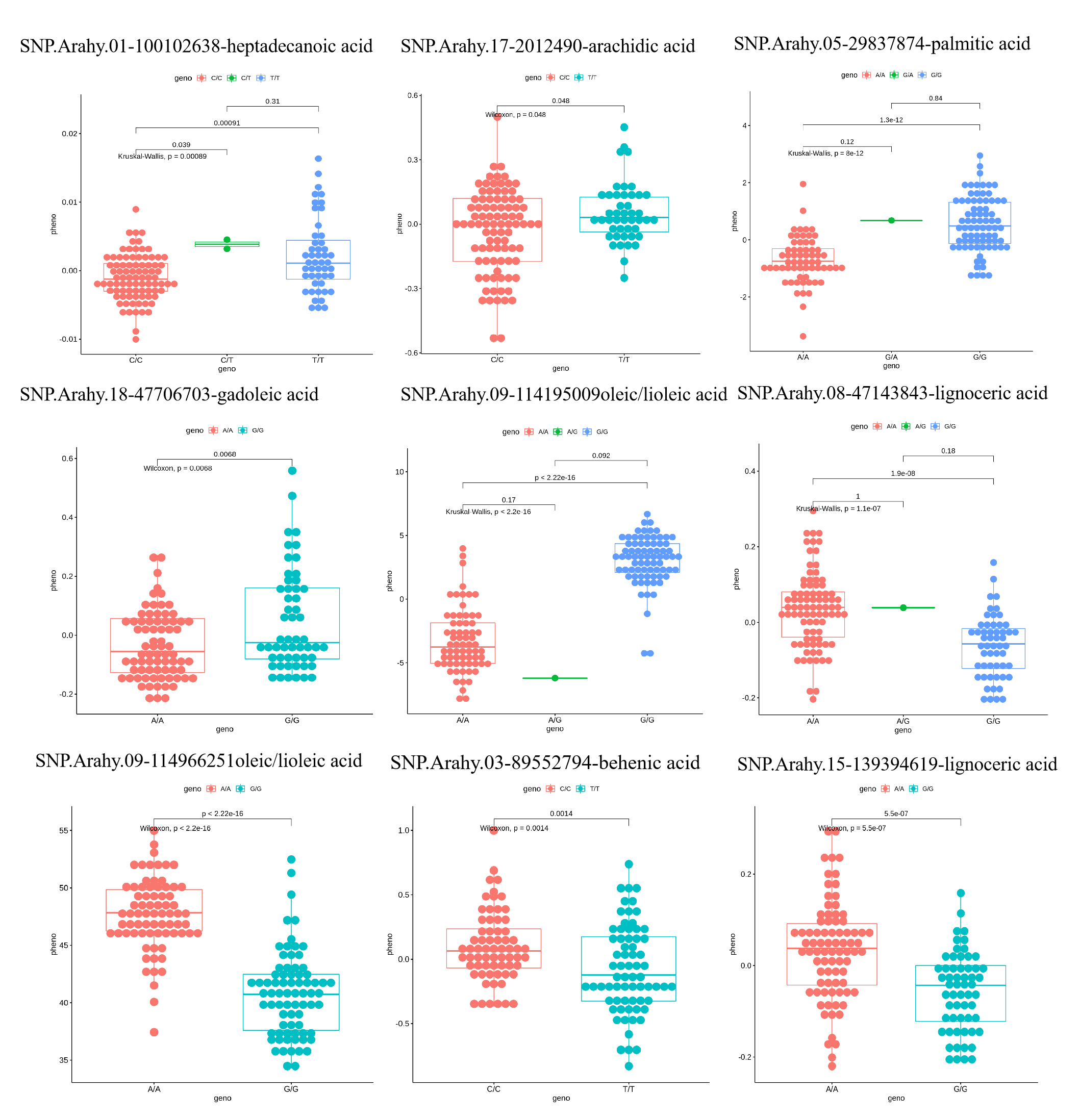

Supplement: Supplementary file 1 [file plants-13-00016-s001.zip › plants-2749434-supplementary/Figure S2.png]
